# Why digital innovation may not alleviate healthcare’s environmental impacts

**DOI:** 10.1136/bmj-2023-078303

**Published:** 2024-06-03

**Authors:** Gabrielle Samuel, Geoffrey M Anderson, Federica Lucivero, Anneke Lucassen

**Affiliations:** 1 Department of Global Health and Social Medicine, King’s College London, London, UK; 2 Institute of Health Policy, Management and Evaluation, University of Toronto; 3 Ethox Centre, University of Oxford, UK; 4 Clinical Ethics, Law, and Society Group, Wellcome Centre for Human Genetics, University of Oxford, Oxford, UK; 5 Centre for Personalised Medicine, University of Oxford, Oxford, UK

## Abstract

In order for digital innovations to have a positive role in efforts to make healthcare more environmentally sustainable, it is important to understand the environmental consequences of investment in digital infrastructure, argue Samuel and colleagues.

## Introduction

Healthcare is becoming increasingly digitalised through innovations in information and communication technologies, including monitoring devices, streaming, data storage, and increasingly through advances in machine learning and artificial intelligence (AI) (1). Advocates enthuse that this digitalisation will improve key aspects of healthcare delivery, including enhanced safety, accessibility, quality of care, effectiveness, and efficiency (2). Others debate whether these promises can be met because of complex social, cultural, economic, and political implementation challenges (3). More recently, digital innovation has been promised as a means to reduce the environmental impacts associated with healthcare delivery (4). Healthcare systems contribute to approximately 5.5% of a country’s total greenhouse gas emissions, with this figure often being higher in high income countries (HICs) (5). In this paper we argue that digitalisation could indeed reduce environmental impacts, but that it is also possible that these technologies might be implemented in ways that do not lead to reductions, and indeed may increase resource use with little change to health outcomes. We discuss current evidence that shows how digital innovation could reduce the environmental impacts of healthcare. Then we focus on two aspects that need to be considered when developing plans for a digitally enabled and environmentally sustainable healthcare system, namely the growing energy- and resource-intensive digital infrastructures required, and the paradoxical increase in energy use associated with the introduction of energy-saving technologies–the so called “rebound effect”. Our perspective predominantly reflects HIC health systems, though our arguments are relevant globally, and we also draw on and discuss low and middle income country (LMIC) contexts.

## Evidence that digital innovation reduces healthcare’s environmental impacts

Some of the ways in which digital innovations might decrease the environmental impacts of health systems are described in Table 1.

## How digital tools might/can reduce healthcare’s environmental impacts

First, digital innovations are expected to help reduce the environmental impacts associated with existing healthcare facilities by improving the efficiency of operations. The UK National Health Service (NHS) has predicted carbon savings through the use of real-time monitoring, including artificial intelligence, to better control buildings (e.g., controlling lights, heating, and cooling) and to forecast resource allocation more effectively (6). Digital technologies have also been shown to be valuable in predicting electricity and water consumption across various healthcare facilities so that focussed attention can be provided when consumption varies, to examine and address the causes (7).

Second, digital innovations are expected to provide a substitute for services and/or processes that use materials and energy. For example, digital technologies have helped reduce paper use in the development of electronic medical records, and chemical use for film in digital imaging infrastructures. Telemedicine reduces transport-related emissions when compared to equivalent in-person appointments (8). Remote monitoring of out-patients can also reduce transport-associated carbon emissions (9, 10). Remote outpatient monitoring also promises to reduce energy and material use through the earlier detection of clinically important symptoms, thereby reducing the need for intensive healthcare intervention (11, 12).

Third, the increasing move to telemedicine and remote monitoring is expected to mean that fewer people will attend hospitals which will mean smaller healthcare facilities with lower carbon emissions (13). Pilot studies already suggest the effectiveness of remote real-time monitoring of patients’ blood pressure (14), and blood glucose levels (15). Preliminary evidence also suggests digitally delivered therapy can be effective in helping support those struggling with their mental health (16, 17). The application of AI as a tool to improve the effectiveness and accessibility of health care delivery is well underway in HICs and of growing interest in LMICs (18).

At the same time, some research is showing evidence in a different direction–that of increased consumption patterns associated through telemedicine. *Quality Watch*–a joint research programme for the UK Health Foundation and UK Nuffield Trust–found that more follow-up appointments were required and there was a lower rate of discharge from a service for online compared to face-to-face appointments. Moreover, online appointments led to increased rates of new prescriptions and referrals (19). Further complexities include ensuring patient privacy during virtual appointments, and internet access problems, both of which may mean follow-up face-to-face appointments are arranged in addition. In turn these may affect disadvantaged socio-economic groups disproportionately, leading to widening inequities.

## Growth in Digital Infrastructure

Although generally positioned as reducing environmental impacts, digital technologies themselves have emissions from the energy used to collect, store, process, and analyse data. Greenhouse emissions associated with digital technologies are difficult to quantify and will depend on (amongst other factors) when and where data are stored and processed. Estimates suggest that the digital sector accounts for between 1.8-2.8% of all global emissions (20). The carbon footprint of storing and processing data depends on numerous factors (geographies; type of storage; server used, and more), but is thought to be around ~10 kg CO2e (range 4-28kg) for 1 terabyte of data storage per year (21), and will rise with increased storage. The magnitude of this rise depends on the use of renewables, types of data storage, and hardware and software efficiencies.

Over the next few years, healthcare is primed to grow faster than other sectors in the digital sphere because of advancements in healthcare analytics, with some industry predictions stating that it will exceed 10 zettabytes (ten trillion gigabytes) by 2025 (22). Advancements in digital pathology offer a useful example of such growth, with clinically applicable deep-learning support already available in cancer pathology, and a German study estimating that deep learning led analysis of worldwide pathology cases would be contribute about 16 megatons of carbon dioxide per year (23) (this is equivalent to an average gasoline-powered passenger vehicle being driven 40921 miles per year). A recent commentary on the use of AI for imaging and informatics highlighted the rapidly increasing computational intensity of AI models, raised sustainability concerns, and argued for full life-cycle assessment of impacts of AI models (24). Developments in genomics provide another good example. Several NHS-embedded research studies offer whole genome sequencing (WGS), for example the imminent newborn WGS screening study. Whilst only a tiny proportion of the genome (often <0.01%) will be analysed to screen for genetic conditions and informing the newborn’s current healthcare (25), the rest of the genomic data will be stored, and the storage costs for WGS are significantly greater than hitherto targeted genetic testing. The fact that these ventures straddle both research and health-care settings–and indeed the drive for WGS is research led–means the footprint for healthcare is difficult to measure, but the WGS of half a million participants in UK Biobank requires about 27.5 petabytes of storage (26).

The manufacture, use, and disposal of digital technologies also have other environmental impacts. For example, the environmental cost of mining- and depleting-natural resources; the use of water to provide systems to cool digital servers during use, and the disposal of electronic/hardware waste (27–29). These practices often rely on extractivist and exploitative practices, which particularly affect individuals and communities in LMICs (30). For example, electronic waste is often disposed of in LMICs where it offers a route to income through informal recycling practices. However, this informal work is part of broader neo-colonial processes that can amplify, or widen inequities, and furthermore, these practices, such as burning and acid bathing, often lead to health harms to those working and living in the vicinities (31).

Over the last half a decade, improvements in digital capabilities have allowed for increasing efficiency so that energy and resource consumption has not increased in line with societies’ growing appetite to gather and process evermore data. However, efficiency gains will likely no longer be enough to offset the drive to create and gather ever for more data.

## Rebound effect

The energy policy sector has demonstrated that behaviour often changes in response to perceived cost and energy savings, and this can lead to energy savings being less than expected. This phenomenon is called a rebound effect (32), and one that increases energy usage overall is called backfire. A highly efficient refrigerator may still consume more energy than a small G rated one, because–thinking it is efficient–we might open the door more, clean it less, and/or purchase additional appliances with the savings. Equally, in the health care setting, if we use any savings made to collect and process more data, overall savings reduce. Health and social care systems in England have recently moved data to a Cloud based server to improve storage and transmission (33), but this may have the effect of allowing the collection and processing of evermore data–for example, gathering WGS routinely rather than clinically guided genetic testing. Remote monitoring may also lead to increased digital consumption because more powerful energy- and resource-intensive algorithms are utilised on patient data. This is particularly the case for digital phenotyping where data from smart devices create an holistic digital picture of behaviours, often utilising AI (34, 35). Google has reported that AI represents 10-15% of their power use (36).

## Moving forward

Some of the concerns outlined above may be addressed with moves towards renewable energy. Health systems and facilities in industrialised countries are already moving towards the use of renewable energy (37), and in LMICs, there are many examples of new health systems being developed alongside renewable infrastructures to ensure energy sufficiency (38). Furthermore, many large technology companies providing Cloud services to the healthcare sector have already declared net zero targets (39).

However, despite the digital sector’s support of renewable energy, this does not mean growth of this sector has no environmental consequences. For instance, data centres may outrun renewable power consumption and/or leave a shortfall for other sectors (20). Furthermore, many healthcare systems in LMICs remain fossil-fuel powered, especially in countries where electricity outages and the need for back-up generators are common (40). Overall, this is not a reason to refrain from digital technology use, especially if clear patient benefit exists. What is important, though, is that this patient benefit is *assessed* rather than *assumed*, and that digital technologies are implemented in ways that mitigate their associated environmental harms as much as possible.

To do this, efficiencies gained through the use of digital technologies must not be seen as a fix to addressing the environmental impacts of healthcare systems, but rather, as one tool in the toolbox to making healthcare more environmentally sustainable (41). In this toolbox, monitoring and evaluating the positive and negative environmental and health impacts of digitalisation is important. (It is of course worth noting that any evaluation will itself require data, which has its own environmental impact.) While such assessments are complex because digital pathways are often lacking from databases that collate information about emissions associated with healthcare activities (42), such assessments are necessary tools and should evolve to include measurement of rebound effects through more deliberative qualitative and quantitative reflection (43). The SusQI framework, developed by the UK Centre for Sustainable Healthcare, is one attempt to do this, which while not specific for digitalisation, is a good starting point (44).

Furthermore, assessment frameworks need to embed into broader international and local digital health governance structures such as WHO’s recent strategy (45) or NICE evidence standards (46) so they can bring attention to environmental issues alongside other considerations, such as safety, equitability, and effectiveness. They also need to be embedded into procurement strategies for digital software, hardware, and services (47). The importance of context is important here because countries will differ in how best they can consider environmental implications given competing (health) priorities, as well as other potential constraints (for instance resources and data sovereignty issues), and these differences may be starker between HICs and LMICs.

## Conclusion

Healthcare systems face the daunting challenge of rapidly decarbonizing and becoming environmentally sustainable as part of the global response to climate change. Although many hope the ongoing digitalisation of healthcare is a promising pathway to healthcare system sustainability, we suggest that getting to that goal is nuanced and complex. More comprehensive ways to measure and understand the environmental impacts associated with the digitalisation of healthcare systems are needed. There also needs to be a more nuanced informed debate about assumptions that more data and advanced data processing must be a good thing for healthcare. Such considerations need to sit within wider digital health governance frameworks to bring legitimacy to decision-making.

## Figures and Tables

**Figure:**
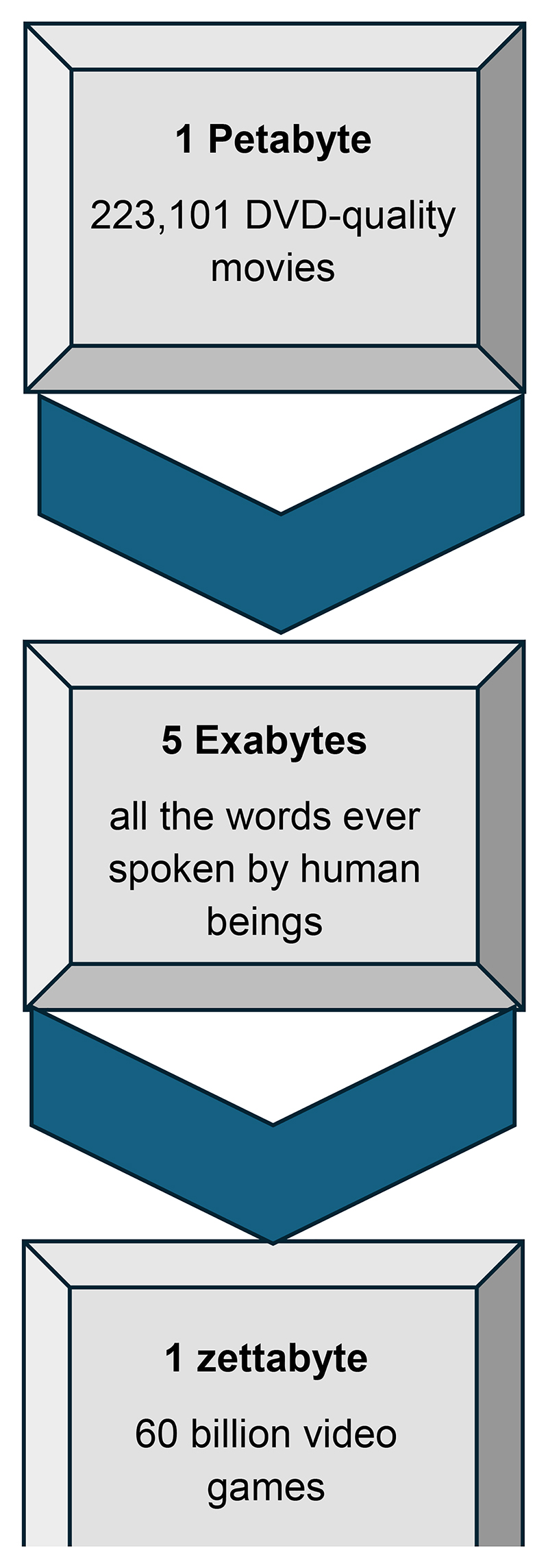


**Table 1:** Examples of some of the ways in which digital technologies are promised to reduce the negative environmental impacts associated with healthcare.

How digital tools might/can reduce healthcare’s environmental impacts	Examples
Improving the operational efficiency of existing healthcare infrastructure	Use sensors for turning off lights and controlling room temperatures Forecasting healthcare facility energy and water consumption to detect and address anomalies Forecasting resource use so only the necessary supplies are purchased
Providing applications and/or services that have lower environmental impacts than non-digital ones	Replacing paper records with electronic medical records Replacing in-person visits with virtual visits
Keeping the population healthy and reducing the demand for healthcare	Using large data bases and advanced AI algorithms to support improved clinical decision making and patient interaction Employing advanced analytics to forecast utilisation and manage inventories and personnel efficiently

## Key Messages

- The digitalisation of healthcare involves a range of information and communication technologies, including growing investments in computationally intense research with large datasets and artificial intelligence innovations.
- Digital innovations have the potential to play a central important role in efforts to reduce the environmental impacts of healthcare.
- At the same time, such innovations have environmental impacts. Consideration of these impacts is now crucial if they are to be used in a way that benefits both the environment and health.

## References

[1] Fatehi F, Samadbeik M, Kazemi A (2020). What is Digital Health? Review of Definitions. Stud Health Technol Inform.

[2] Topol E (2019). Preparing the healthcare workforce to deliver the digital future.

[3] Petersen A (2018). Digital Health and Technological Promise.

[4] Patel S (2021). The role of digital technologies in meeting NHS net zero targets.

[5] Pichler P-P, Jaccard IS, Weisz U, Weisz H (2019). International comparison of health care carbon footprints. Environmental Research Letters.

[6] NHS (2020). Delivering a ‘Net Zero’ National Health Service.

[7] Peng Y, Zhang M, Yu F, Xu J, Gao S (2020). Digital Twin Hospital Buildings: An Exemplary Case Study through Continuous Lifecycle Integration. Advances in Civil Engineering.

[8] Purohit A, Smith J, Hibble A (2021). Does telemedicine reduce the carbon footprint of healthcare? A systematic review. Future Healthc J.

[9] Bawa D, Ahmed A, Darden D, Kabra R, Garg J, Bansal S (2023). Impact of Remote Cardiac Monitoring on Greenhouse Gas Emissions: Global Cardiovascular Carbon Footprint Project. JACC: Advances.

[10] Moses R, Taylor C, Wood S, Vyas A (2016). P191 Reducing the carbon footprint in a regional long term ventilation service with the use of remote monitoring. Thorax.

[11] Imberti JF, Tosetti A, Mei DA, Maisano A, Boriani G (2021). Remote monitoring and telemedicine in heart failure: implementation and benefits. Current Cardiology Reports.

[12] Islam MR, Kabir MM, Mridha MF, Alfarhood S, Safran M, Che D (2023). Deep Learning-Based IoT System for Remote Monitoring and Early Detection of Health Issues in Real-Time. Sensors.

[13] Simms N, Beausejour W, Gheorghiu B (2022). The environmental benefits of virtual care utilization in Canada: An analysis of travel distance avoided and associated carbon reductions as reported in the Canada Health Infoway Canadian Digital Health Survey 2021: What Canadians Think. White Papers.

[14] McManus RJ, Little P, Stuart B, Morton K, Raftery J, Kelly J (2021). Home and Online Management and Evaluation of Blood Pressure (HOME BP) using a digital intervention in poorly controlled hypertension: randomised controlled trial. BMJ.

[15] Amante DJ, Harlan DM, Lemon SC, McManus DD, Olaitan OO, Pagoto SL (2021). Evaluation of a Diabetes Remote Monitoring Program Facilitated by Connected Glucose Meters for Patients With Poorly Controlled Type 2 Diabetes: Randomized Crossover Trial. JMIR Diabetes.

[16] Hollis C, Hall CL, Jones R, Marston L, Novere ML, Hunter R (2021). Therapist-supported online remote behavioural intervention for tics in children and adolescents in England (ORBIT): a multicentre, parallel group, single-blind, randomised controlled trial. The Lancet Psychiatry.

[17] Fossey J, Charlesworth G, Fowler J-A, Frangou E, Pimm TJ, Dent J (2021). Online Education and Cognitive Behavior Therapy Improve Dementia Caregivers’ Mental Health: A Randomized Trial. Journal of the American Medical Directors Association.

[18] Schwalbe N, Wahl B (2020). Artificial intelligence and the future of global health. Lancet.

[19] Quality Watch (2020). The remote care revolution during Covid-19.

[20] Freitag C, Berners-Lee M, Widdicks K, Knowles B, Blair GS, Friday A (2021). The real climate and transformative impact of ICT: A critique of estimates, trends, and regulations. Patterns.

[21] Lannelongue L, Aronson H-EG, Bateman A, Birney E, Caplan T, Juckes M (2023). GREENER principles for environmentally sustainable computational science. Nature Computational Science.

[22] Consulting LEK (2023). Tapping Into New Potential: Realising the Value of Data in the Healthcare Sector.

[23] Vafaei Sadr A, Bülow R, von Stillfried S, Schmitz NEJ, Pilva P, Hölscher DL (2024). Operational greenhouse-gas emissions of deep learning in digital pathology: a modelling study. The Lancet Digital Health.

[24] Jia Z, Chen J, Xu X, Kheir J, Hu J, Xiao H (2023). The importance of resource awareness in artificial intelligence for healthcare. Nature Machine Intelligence.

[25] Horton R, Wright CF, Firth HV, Turnbull C, Lachmann R, Houlston RS (2024). Challenges of using whole genome sequencing in population newborn screening. BMJ.

[26] UK Biobank (2023). Your Genetics, Everybody’s health: Annual Newsletter 2023/24.

[27] Doron A, Jeffrey R (2018). India’s unofficial recycling bin: the city where electronics go to die. Guardian.

[28] Lizarraga C, Solon O (2023). Thirsty Data Centers Are Making Hot Summers Even Scarier.

[29] Mancini L, Eslava NA, Traverso M, Mathieux F (2021). Assessing impacts of responsible sourcing initiatives for cobalt: Insights from a case study. Resources Policy.

[30] Frazzoli C, Ruggieri F, Battistini B, Orisakwe OE, Igbo JK, Bocca B (2022). E-WASTE threatens health: The scientific solution adopts the one health strategy. Environmental Research.

[31] Little P (2021). Burning Matters: Life, Labor, and E-Waste Pyropolitics in Ghana.

[32] Hilty LM, Köhler A, Von Schéele F, Zah R, Ruddy T (2006). Rebound effects of progress in information technology. Poiesis & Praxis.

[33] NHS Digital Spine.

[34] Kourtis LC, Regele OB, Wright JM, Jones GB (2019). Digital biomarkers for Alzheimer’s disease: the mobile/wearable devices opportunity. npj Digital Medicine.

[35] Jayakumar P, Lin E, Galea V, Mathew AJ, Panda N, Vetter I (2020). Digital Phenotyping and Patient-Generated Health Data for Outcome Measurement in Surgical Care: A Scoping Review. J Pers Med.

[36] Anderson J, Sweeney D, Canonica R (2023). POWER OF AI: Wild predictions of power demand from AI put industry on edge.

[37] The renewable energy hub Sustainable Future for UK Hospitals with Solar Energy.

[38] Associates A HETA: Powering Health with Clean Energy in Africa.

[39] Amazon (2022). Reaching Net Zero Carbon by 2040.

[40] World Health Organisation (2023). Energizing health: accelerating electricity access in health-care facilities.

[41] Widdicks K, Lucivero F, Samuel G, Croxatto LS, Smith MT, Holter CT (2023). Systems thinking and efficiency under emissions constraints: Addressing rebound effects in digital innovation and policy. Patterns.

[42] Drew J, Christie SD, Rainham D, Rizan C (2022). HealthcareLCA: an open-access living database of health-care environmental impact assessments. The Lancet Planetary Health.

[43] Smith CL, Zurynski Y, Braithwaite J (2022). We can’t mitigate what we don’t monitor: using informatics to measure and improve healthcare systems’ climate impact and environmental footprint. Journal of the American Medical Informatics Association.

[44] Frances M, Jennifer I, Alexander W, Emma V (2018). Sustainability in quality improvement: redefining value. Future Healthcare Journal.

[45] WHO (2021). Global strategy on digital health 2020-2025.

[46] National Institute for Health and Care Excellence (2022). Evidence standards framework for digital health technologies.

[47] Carr K, Clark A, Matthews M (2024). Building a Sustainable ICT Ecosystem: Strategies and Best Practices for Reducing Environmental Harms in a Digital World.

